# Multi‐omics predictive model based on clinical, radiomic and genomic features for predicting the response of limited‐stage small cell lung cancer to definitive chemoradiotherapy

**DOI:** 10.1002/ctm2.1522

**Published:** 2024-01-09

**Authors:** Li Li, Jinghao Duan, Yongsheng Gao, Ying Yin, Fengchang Yang, Wenjie Tang, Xiaoyu Song, Tao Hu, Jinfeng Cui, Jinming Yu, Shuanghu Yuan

**Affiliations:** ^1^ Department of Radiation Oncology Shandong Cancer Hospital and Institute Shandong First Medical University and Shandong Academy of Medical Sciences Jinan Shandong China; ^2^ Department of Pathology Shandong Cancer Hospital and Institute Shandong First Medical University and Shandong Academy of Medical Sciences Jinan Shandong China; ^3^ Department of Oncology Second People's Hospital of Yibin City Yibin Sichuan China; ^4^ Department of Radiology Shandong Cancer Hospital and Institute, Shandong First Medical University and Shandong Academy of Medical Sciences Jinan Shandong China; ^5^ Department of Radiation Oncology The Affiliated Cancer Hospital of Zhengzhou University Henan China

Dear Editor,

The efficacy of definitive concurrent or sequential chemoradiotherapy (dCRT) varies significantly among limited‐stage small‐cell lung cancer (LS‐SCLC) patients, with about 10%–13% of patients achieving 5‐year survival, while 58% of patients die within 1 year.^1^, ^2^, ^3^ Therefore, there is an urgent need to find biomarkers for early prediction of the efficacy of dCRT in LS‐SCLC in support of risk stratification. Tumourigenesis and progression are heterogeneous at the phenotypic, physiologic and genomic levels, making predictive information obtained via radiomic or genomic profiling alone of limited value for clinical decision making.^4^ **^‐^** ^6^ The present study aimed to develop a combination of genomic, radiomic and fused radiogenomic biomarkers for predicting the response of LS‐SCLC to dCRT in training and validation cohorts, and to provide optimised multi‐omics prediction models based on their predictive power for LS‐SCLC.

Totally 154 patients with LS‐SCLC who received dCRT in Shandong Cancer Hospital and Institute were included, and were randomly divided into a training group and test group at a ratio of 7:3. No significant differences in clinical or genomic characteristics were found between the two cohorts (Table S1). The median PFS (progression free survival, mPFS) among all patients was 12.7 months (range, 2.4−60.5 months).

In the training cohort, LASSO regression^7^ was performed to obtain the most significant radiomic features related to PFS according to a *λ*_min_ of .046 (Figure 1A,B). The radiomic signature (Rad‐score) was then constructed by linearly combining the 10 selected features and corresponding weighting coefficients, as listed in Table S2. The best threshold was .35,^8^ which divided patients into a high‐risk group (Rad‐score ≥ .35) and a low‐risk group (Rad‐score < .35). Rad‐score was identified as an independent biomarker for PFS on both univariate and multivariate Cox analyses. The correlation between Rad‐score and PFS was significant in the training cohort (mPFS, 14.83 vs. 10.63 months, *p* = .006; hazard ratio = 2.152, 95% confidence interval: 1.236−3.749, *p* = .007), as shown in Figure 2 and Table S3. The *C*‐index for the ability of the Rad‐score to predict PFS in the training set was .574, and the area under the curve (AUC) values for prediction of 6‐ and 12‐month PFS were .583 and .601, respectively (Figure 3 and Table S4). A significant association between Rad‐score and PFS was also demonstrated in the validation cohort (mPFS, 14.20 vs. 7.83 months, *p* = .015; *C*‐index = .656; AUC for 6‐ and 12‐month PFS: .746 and .640, respectively).

**FIGURE 1:**
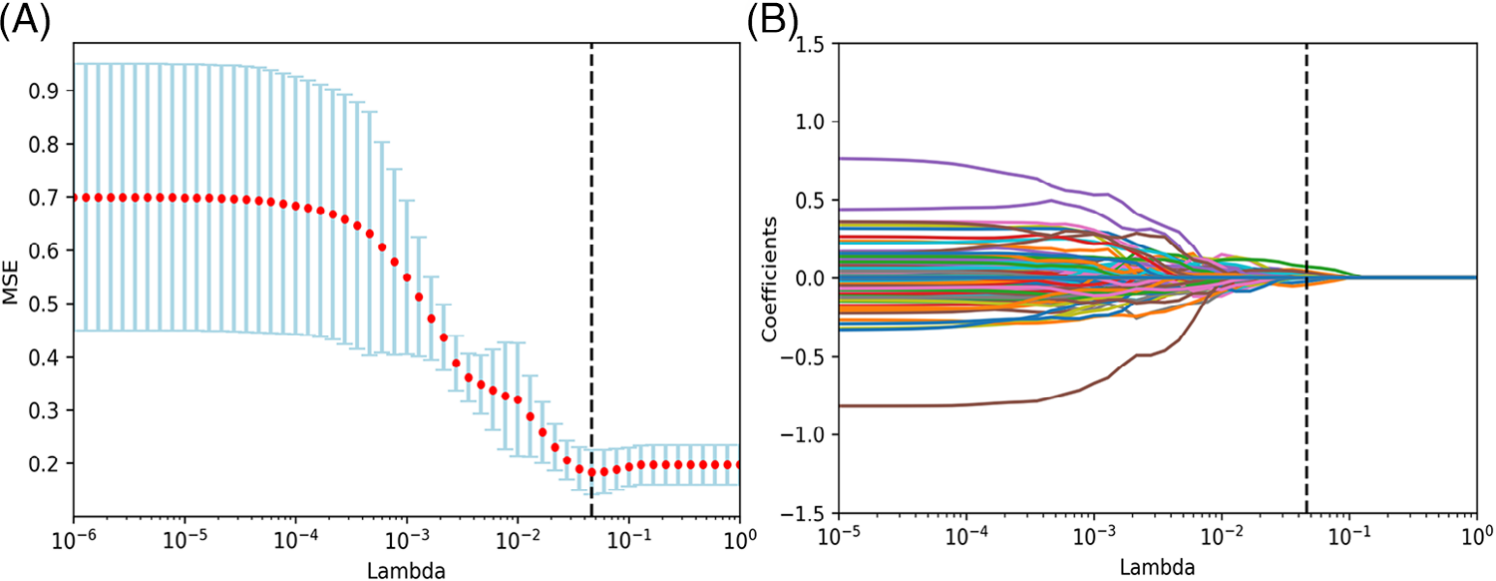
Selection of radiomic features related to PFS using LASSO regression. (A) Cross‐validation curve. (B) Coefficients curves for radiomic features. MSE, mean square error.

**FIGURE 2:**
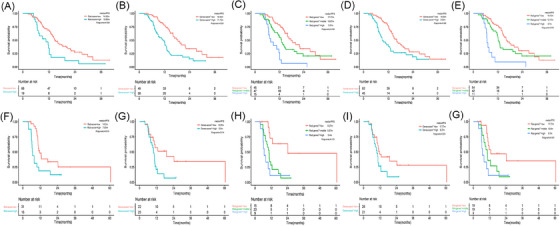
Survival analyses of Rad‐score (A and F), Genes‐score^pr^ (B and G), Rad‐Genes^pr^ (C and H), Genes‐score^po^ (D and I) and Rad‐Genes^po^ (E and G) in the training and validation cohorts.

**FIGURE 3:**
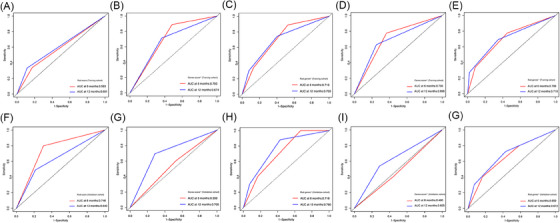
The receiver operating characteristic (ROC) curve analyses of Rad‐score (A and F), Genes‐score^pr^ (B and G), Rad‐Genes^pr^ (C and H), Genes‐score^po^ (D and I) and Rad‐Genes^po^ (E and G) in the training and validation cohorts.

We previously identified novel biomarkers of alterations in the *CDK4*, *GATA6* and MAPK/ERK pathway genes as well as tumour mutational burden (TMB) status as predictors of the response to dCRT in a large cohort of LS‐SCLC patients.^9^ According to the prior genomic model (Genes‐score^pr^) combined by these four features, patients with low Genes‐score^pr^ (no gene mutations and high TMB) showed significant improved PFS in training and validation cohorts (mPFS, low Genes‐score^pr^ vs. high Genes‐score^pr^, 18.43 vs. 11.13 months, *p* < .001; mPFS, low Genes‐score^pr^ vs. high Genes‐score^pr^, 9.27 vs. 5.8 months, *p* = .014) (Figure 2).

And, posterior genomic biomarkers (Genes‐score^po^) of *CDK4* and TMB status were recognised as significant factors (Table S3) according to the univariate and multivariate Cox analyses. In the training group, patients with a low Genes‐score^po^ (no *CDK4* amplification and high TMB) showed significantly prolonged PFS compared with patients with high Gene‐score^po^ (*CDK4* amplification and/or low TMB) (mPFS, 16.03 vs. 9.03 months, *p* = .006) (Figure 2). In the validation set, Kaplan–Meier analysis showed that the Genes‐score^po^ model could effectively distinguish SCLC patients with different PFS durations (mPFS, low Genes‐score^po^ vs. high Genes‐score^po^, 17.77 vs. 9.27 months, *p* = .001) (Figure 2).

As shown in Figure 3 and Table S4, the corresponding combination of radiogenomic models (Rad‐Genes^pr^ and Rad‐Genes^po^) all demonstrated higher *C*‐index and 6‐ and 12‐month AUC values of the ability to predict PFS than individual radiomic (Rad‐score) or genomic models (Genes‐score^pr^ and Genes‐score^po^), respectively.

To the best of our knowledge, no research has been conducted to date to determine the ability of fused radiogenomic features to predict the efficacy of dCRT in LS‐SCLC.^10^ According to the Rad‐score, Genes‐score^pr^ and Genes‐score^po^, Kaplan–Meier analyses were conducted according to the combination signature (Rad‐Genes^pr/po^) built from the radiogenomic factors (Figure 2). Significant associations (log‐rank *p* < .05) were found between Rad‐Genes^po^ and PFS in the training and validation subgroups. The mPFS durations for the high‐risk, intermediate‐risk and low‐risk groups were 6.70, 12.17 and 16.10 months, respectively, in the training cohort and 6.7, 10.5 and 17.77 months, respectively, in the validation cohort. However, Rad‐Genes^pr^ model was only associated with PFS in the training cohort (mPFS, 17.77 vs. 13.07 vs. 7.67 months, *p* < .001), there was no statistical difference in the validation cohort (mPFS, 9.27 vs. 5.87 vs. 5.4 months, *p* = .121).

Overall, we identified several radiomic, genomic and radiogenomic biomarkers with the potential to identify LS‐SCLC patients with reduced risk of progression after dCRT, and a combination of radiogenomic features was found to form the optimal prediction model based on the higher *C*‐index and AUC values compared with individual radiomic and genomic models. Given that the Rad‐Genes^pr^ model failed to show a survival difference in the validation cohort, Genes‐score^po^ developed by CDK4 and TMB maybe better genomic models. The radiogenomic model combining the Rad‐score model, *CDK4* amplification and TMB status could successfully stratify patients into high‐risk, intermediate‐risk and low‐risk groups, and thus, may be conducive for screening SCLC patients according to the likelihood of improved PFS. As our research was conducted by retrospective, single centre and relatively small sample size of patients, which may limit the generalisability of the results. And the combined radiogenomic predictive model established in this study requires external validation with a larger sample size of data collected from more medical centres.

## AUTHOR CONTRIBUTIONS

Li Li designed this study. Li Li and Ying Yin acquired clinical data and performed patient follow‐ups. Li Li, Jinghao Duan, Yongsheng Gao and Fengchang Yang performed data analysis. Li Li, Wenjie Tang, Xiaoyu Song, Jinfeng Cui and Tao Hu edited the manuscript. Jinming Yu and Shuanghu Yuan conceived and supervised the study.

## CONFLICT OF INTEREST STATEMENT

The authors declare they have no conflicts of interest.

## CONSENT FOR PUBLICATION

All authors read and approved the final manuscript.

## ETHICS STATEMENT

The study was approved by the Ethics Committee of Shandong Cancer Hospital and Institute (No. SDTHEC2020004042). Written informed consent was obtained from each patient before sample collection.

## Supporting information

Supporting information

## Data Availability

The datasets generated and/or analysed during this study are available from the corresponding author upon reasonable request.
