# Supplementary material for: Multi‐omics predictive model based on clinical, radiomic and genomic features for predicting the response of limited‐stage small cell lung cancer to definitive chemoradiotherapy
Source: Clin Transl Med. 2024 Jan 9;14(1):e1522. doi: 10.1002/ctm2.1522 (PMC10775182; doi:10.1002/ctm2.1522)
Supplement: Supplementary file 1 — Supporting information [file CTM2-14-e1522-s001.docx]

**Materials and Methods**

**Patients**

This retrospective study included 154 patients with LS-SCLC who received dCRT in Shandong Cancer Hospital and Institute. Patients with a history of systemic treatment or radiotherapy for thoracic cancers or other concomitant cancers were ineligible. This study was approved by the Ethical Committee of Shandong Cancer Hospital and Institute (No. SDTHEC2020004042).

**Data collection and model construction**

Patients were randomly divided into a training group and test group at a ratio of 7:3. Genomic, radiomic and multi-omic (clinical, radiomic and genomic features) feature selection and model building were performed from the training cohort data, and the model was validated using the test cohort data.

***Image data collection and radiomic prediction model construction***

Baseline CT images of patients were acquired. Regions of interest (ROIs) were delineated by experienced radiologists based on the size, shape, and margin of tumor lesions using 3D-Slicer software (version 4.11). All radiomic features were calculated using the PyRadiomics tool version 3.0, referring to an open-source python package from medical images. In total, 851 radiomic features were extracted by PyRadiomics from each patient, including 14 shape features (shape), 18 first-order statistics (first-order), 75 texture features, and 744 higher-order features.

All extracted features were normalized using the Z-score to ensure comparability. To determine intra-observer and inter-observer repeatability, ROIs of 21 patients were reoutlined by different radiologists, and features with an interclass correlation coefficient >0.75 were considered to have good reproducibility. The optimum parameter lambda (λ) was selected from the least absolute shrinkage and selection operator (LASSO) model using 10-fold cross validation to select the significant features associated with progression-free survival (PFS), and the radiomic model (Rad-score) was established in the training cohort^1^. Differences were analyzed with a cutoff value determined by X-tile software^2^.

***Genomic analyses and genomic prediction model construction***

Formalin-fixed paraffin-embedded (FFPE) sections with at least 10% tumor content were collected for NGS. Genomic DNA extraction, library preparation and NGS data analysis were performed as previously described^3^. According to the genomic biomarkers (*CDK4*, *GATA6*, MAPK/ERK pathway genes, and TMB status) of post-dCRT recurrence risk, the combined approach was added to formulate the prior genomic model (Genes-score^pr^). Also, the posterior genomic biomarkers (Genes-score^po^) were combined by analyzing the correlations between these four biomarkers and PFS according to univariate regression analysis.

***Multi-omic predictive model construction***

For building the multi-omic prediction model from clinical, radiomic, and genomic features, the established Rad-score, Gene-score and other clinical features (e.g., sex, age, history of smoking, tumor site, radiotherapy dose, dose fractionation, therapeutic regimen, radiotherapy method) were all included in a univariate Cox regression analysis, and all statistical features were further analyzed by multivariate Cox regression analysis. The significant Rad-score, Genes-score^pr^ and Genes-score^po^ features were combined together to establish the corresponding radio-genomic (Rad-genes) model. With assigning values into the high or low group, the combinatorial Rad-genes model of Rad-score and Genes-score^pr^ or Genes-score^po^ revealed 3 distinct risk groups, respectively. Total 6 model integrating both radiomics and genomic features (Rad-genes^pr^=low, Rad-genes^pr^=middle, Rad-genes^pr^=high; Rad-genes^po^=low, Rad-genes^po^=middle, Rad-genes^po^=high;) were constructed and compared.

***Statistical analysis***

All statistical analyses were performed using R software (version 4.1.2). The differences in clinical and treatment information were assessed by Chi-square test. The potential association of each established prediction model with PFS was assessed using Kaplan–Meier analysis and the log-rank method. Receiver operating characteristic (ROC) curve analysis and AUC values were used to evaluate the prediction capacity of each model, and the concordance index (C-index) was used to evaluate the discriminative performance of each model. Statistical tests were two-sided, and P values of <0.05 were considered significant.

**References**

**1.** Tibshirani R. The lasso method for variable selection in the Cox model. *Stat Med.* 1997;16:385-395.

**2.** Camp RL, Dolled-Filhart M, Rimm DL. X-tile: a new bio-informatics tool for biomarker assessment and outcome-based cut-point optimization. *Clin Cancer Res.* 2004;10:7252-7259.

**3.** Li L, Tang S, Yin JC, et al. Comprehensive Next-Generation Sequencing Reveals Novel Predictive Biomarkers of Recurrence and Thoracic Toxicity Risks After Chemoradiation Therapy in Limited Stage Small Cell Lung Cancer. *Int J Radiat Oncol Biol Phys.* 2022;112:1165-1176.

### Supplementary tables

**Table S1. Clinical characteristics of all eligible patients**

| **Factors** | **Training cohort** | **Validation cohort** | P |
| --- | --- | --- | --- |
| Sex |  |  | 0.543 |
| Female | 29 (27.1%) | 15 (31.9%) |  |
| Male | 78 (72.9%) | 32 (68.1%) |  |
| Age |  |  | 0.620 |
| ≤59 years | 50 (46.7%) | 24 (51.1%) |  |
| >59 years | 57 (53.3%) | 23 (48.9%) |  |
| Smoking history |  |  | 0.301 |
| Never-smokers | 45 (42.1%) | 24 (51.1%) |  |
| Former-smokers | 62 (57.9%) | 23 (48.9%) |  |
| Tumor location |  |  | 0.208 |
| Left | 47 (43.9%) | 25 (53.2%) |  |
| Right | 59 (55.1%) | 20 (42.6%) |  |
| Mediastinum | 1 (0.9%) | 2 (4.3%) |  |
| Radiation dose |  |  | 0.315 |
| <60 Gy | 48 (44.9%) | 17 (36.2%) |  |
| ≥60 Gy | 59 (55.1%) | 30 (63.8%) |  |
| RT dosage regimen |  |  | 0.383 |
| 1.5 Gy Twice Daily | 24 (22.4%) | 7 (14.9%) |  |
| 2 Gy Once Daily | 83 (77.6%) | 40 (85.2%) |  |
| Treatment regimen |  |  | 0.584 |
| SCRT | 62 (57.9%) | 25 (53.2%) |  |
| CCRT | 45 (42.1%) | 22 (46.8%) |  |
| Radiation type |  |  | 0.079 |
| 3D-CRT | 25 (23.4%) | 5 (10.6%) |  |
| IMRT | 82 (76.6%) | 42 (89.4%) |  |
| *CDK4* |  |  | 1.000 |
| Mutation | 9 (8.4%) | 3 (6.4%) |  |
| Wild-type | 98 (91.6%) | 44 (93.6%) |  |
| *GATA6* |  |  | 0.437 |
| Mutation | 4 (3.7%) | 3 (6.4%) |  |
| Wild-type | 103 (96.3%) | 44 (93.6%) |  |
| MAPK/ERK pathway |  |  | 1.000 |
| Mutation | 16 (15.0%) | 7 (14.9%) |  |
| Wild-type | 91 (85.0%) | 40 (85.1%) |  |
| TMB status |  |  | 1.000 |
| ≥10 mut/Mb | 66 (61.7%) | 29 (61.7%) |  |
| <10 mut/Mb | 41 (38.3%) | 18 (38.3%) |  |

**Table S2. Radiomic features selected for construction of the Rad-score.**

| **No.** | **Selected feature** | **Weighting coefficient** | **Type** |
| --- | --- | --- | --- |
| f_1_ | original_shape_Elongation | -0.1781498729 | Shape |
| f_2_ | original_ngtdm_Complexity | 0.0000022707 | Texture |
| f_3_ | wavelet-LLH_firstorder_Skewness | 0.0440515587 | Higher-order |
| f_4_ | wavelet-LLH_glszm_LargeAreaLowGrayLevelEmphasis | 0.0000528034 | Higher-order |
| f_5_ | wavelet-LLH_gldm_LargeDependenceLowGrayLevelEmphasis | 0.2665222688 | Higher-order |
| f_6_ | wavelet-LHH_firstorder_Mean | -0.0805739854 | Higher-order |
| f_7_ | wavelet-HHL_glcm_Idn | 2.7848069382 | Higher-order |
| f_8_ | wavelet-HHL_glszm_SizeZoneNonUniformityNormalized | 0.2245948397 | Higher-order |
| f_9_ | wavelet-HHH_glcm_ClusterShade | 0.2330775395 | Higher-order |
| f_10_ | wavelet-LLL_ngtdm_Complexity | 0.0000005733 | Higher-order |

**Table S3. Univariate and multivariate Cox analyses of clinical, radiomic and genomic features significantly associated with PFS**

| **Variables** | **Progression-free survival** | | | |
| --- | --- | --- | --- | --- |
|  | **Univariate analysis** | **P** | **Multivariate analysis** | **P** |
|  | **HR (95%CI)** |  | **HR (95%CI)** |  |
| Gender (female vs. male) | 1.318(0.765-2.272) | 0.320 |  |  |
| Age (≤59 years vs. >59 years) | 0.709(0.438-1.148) | 0.162 |  |  |
| Smoking history (Never- vs. Former-smokers) | 1.545(0.946-2.525) | 0.082 |  |  |
| Tumor site (left lung reference) |  | 0.240 |  |  |
| Right lung | 1.532(0.931-2.521) | 0.093 |  |  |
| Mediastinum | 1.609(0.216-11.981) | 0.642 |  |  |
| Radiotherapy dose. (<60 Gy vs. ≥60 Gy) | 0.779(0.483-1.258) | 0.307 |  |  |
| Segmentation method (1.5 Gy twice/day vs. 2 Gy once/day) | 1.027(0.592-1.783) | 0.924 |  |  |
| Chemoradiotherapy (SDRT vs. CCRT) | 0.898(0.552-1.460) | 0.663 |  |  |
| Radiation therapy regimen (3D-CRT vs. IMRT) | 0.649(0.384-1.094) | 0.105 |  |  |
| *CDK4* (WT vs. mutation) | 3.293(1.544-7.023) | 0.002 | 3.081(1.420-6.687) | 0.004 |
| *GATA6* (WT vs. mutation) | 0.904(0.221-3.702) | 0.889 |  |  |
| TMB (low vs. high) | 0.540(0.331-0.881) | 0.014 | 0.537(0.328-0.879) | 0.013 |
| MAPK pathway (WT vs. mutation) | 1.916(0.956-3.840) | 0.067 |  |  |
| Rad-score (low vs. high) | 2.152(1.236-3.749) | 0.007 | 2.028(1.158-3.553) | 0.013 |

### Abbreviations: WT, wild-type.

| **Table S4. Comparison of prediction efficiency of constructed models in the training and validation cohorts** | | | | | | |
| --- | --- | --- | --- | --- | --- | --- |
| Model | 6-month PFS (AUC) | | 12-month PFS (AUC) | | C-index | |
|  | Training set | Validation set | Training set | Validation set | Training set | Validation set |
| Rad-score | 0.583 | 0.746 | 0.601 | 0.640 | 0.574 | 0.656 |
| Gene-score^pr^ | 0.703 | 0.539 | 0.674 | 0.705 | 0.634 | 0.587 |
| Rad-Gene^pr^ | 0.713 | 0.718 | 0.703 | 0.760 | 0.653 | 0.690 |
| Gene-score^po^ | 0.706 | 0.480 | 0.696 | 0.605 | 0.622 | 0.568 |
| *Rad-Gene^po^* | 0.706 | 0.659 | 0.710 | 0.672 | 0.643 | 0.665 |
